# Dynamical regimes and stability of circular granular ratchets

**DOI:** 10.1038/s41598-017-12588-w

**Published:** 2017-10-05

**Authors:** Patric Müller, Jason A. C. Gallas, Thorsten Pöschel

**Affiliations:** 1 Institute for Multiscale Simulations, Friedrich-Alexander-Universität Erlangen-Nürnberg, D-91052 Erlangen, Germany; 2 Departamento de Física, Universidade Federal da Paraíba, 58051-970 João Pessoa, Brazil; 3 Instituto de Altos Estudos da Para ba, Rua Silvino Lopes 419-2502, 58039-190 João Pessoa, Brazil; 4 Max-Planck-Institut für Physik komplexer Systeme, D-01187 Dresden, Germany; 5 Complexity Sciences Center, 9225 Collins Avenue, Suite 1208, Surfside, FL 33154-3046 USA

## Abstract

Ratchets are simple mechanical devices which combine spatial asymmetry and nonequilibrium to produce counterintuitive transport of particles. The operation and properties of *linear* ratchets have already been extensively explored. However, very little is known about *circular granular* ratchets, startling devices able to convert vertical vibrations into rotations of the device. Here, we report results of systematic numerical investigations of the operational characteristics of circular granular ratchets. Several distinct behaviors are identified and explained in terms of the inner flow fields of the ratchet. All dynamical regimes found are robust and should not be difficult to observe in laboratory experiments.

## Introduction

A curious and counterintuitive phenomenon observed in systems in nonequilibrium and with spatial asymmetry is a net transport of macroscopic particles. Such transport is easily seen when particles are subjected to a spatially asymmetric potential (e.g. a saw-tooth potential) combined with a driving force whose temporal average vanishes (e.g. under sinusoidal tilting or stochastic noise). This transport phenomenon is called *ratchet effect* and the systems displaying it are called *ratchets* ^1–9^.

Ratchets operate in two basic geometrical configurations, linear or circular. Transport phenomena in linear ratchets have been extensively investigated and have a well-documented history stretching over more than a century^10–12^. Linear ratchets were studied at a classical and quantum level, and both from theoretical and experimental points of view^1–4,13^. In contrast, circular ratchets^14^ have not yet been studied in detail.

**Figure 1:**
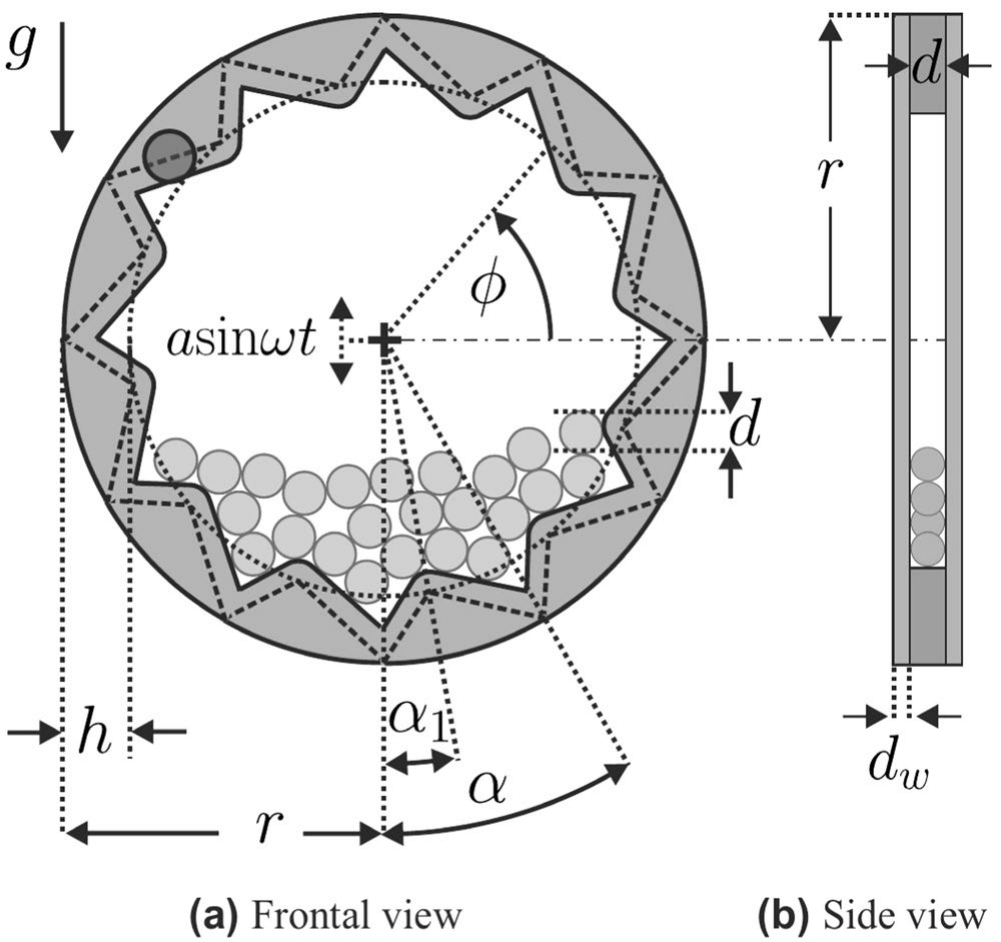
Schematic view of the circular ratchet. The angle *ϕ* measures rotation when the ratchet is vibrated periodically in the vertical direction in an external gravitational field *g*, as indicated. The particle on the left top side is used to construct teeth without discontinuities (see text).

Figure 1 illustrates schematically the circular ratchet that we study here. Such a ratchet is easily obtained by bending the extremities of the familiar linear saw-tooth ratchet such as to form a circle. As demonstrated experimentally, the circular ratchet has the striking ability of converting the chaotic, unordered motion of the particles into directed rotation. The dynamical behavior of circular ratchets is very rich, being sensitive to several physical conditions and control parameters.

The purpose of the present paper is to report results of systematic numerical simulations of the dynamical behavior of a circular ratchet and to study its behavior on the parameters of driving and the internal states assumed by the granular material due to these parameters. A difficulty faced in laboratory experiments is the need of adjusting materials and physical dimensions when sweeping extended parameter intervals. Such variations are more easily implemented and explored in simulations, which can efficiently reveal general trends and appropriate parameter intervals to perform experiments. This is our major motivation for the present study.

## Circular ratchet

Figure 1 shows the circular ratchet investigated here, together with its geometrical characteristics. It consists of a dented circle of external radius *r* containing viscoelastic, frictionless spherical particles. We consider a two dimensional simulation where the motion of the particles is restricted to the plane defined by the dented circle. Only to compute the moment of inertia of the ratchet device, we consider the geometry indicated by the cutaway view in Fig. 1b. Each tooth of the ratchet extends over an angle *α*. The symmetry of every individual tooth is controlled by the angle *α*_1_. We consider *α*_1_ ∈ [0, *α*] where *α*_1_ = *α*/2 corresponds to symmetric teeth. The axis of the ratchet is vibrated periodically in the vertical direction with the time dependent displacement *asin*(*ωt*), as indicated. Gravity points downwards along the vertical direction. To initialize the system, the particles are placed at the center of the ratchet from where they are released to fall down, under the action of gravity, to form a packing at the bottom of the ratchet. Before the sinusoidal driving is activated, we enforce a suitable equilibration phase during which the particles dissipate their kinetic energy completely. Further details of the setup and the simulation method can be found in Sec. *Methods*. A movie illustrating the dynamics of the system can be found at www.mss.cbi.fau.de/sup/ratchet_dynamics.mp4.

## Dynamics of the circular ratchet

**Figure 2:**
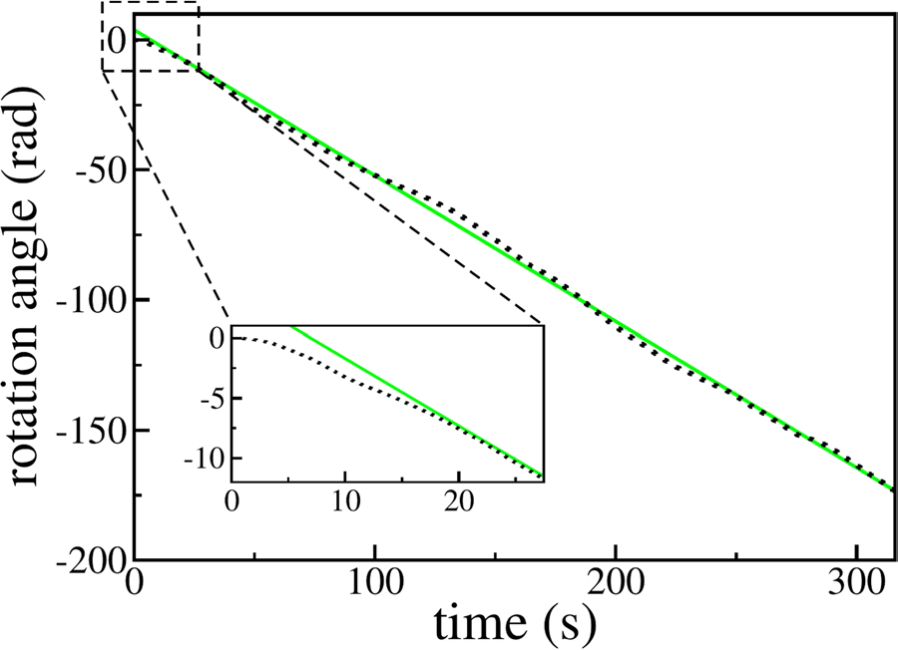
Typical evolution of the rotation angle *ϕ* in the ratchet regime. Negative values indicate clockwise rotation (see Fig. 1). Black dots show the simulation data, the green (grey) line shows a linear regression of the simulation data: *ϕ*(*t*) = 3.91 − 0.56 s^−1^ *t*. The inset shows a magnification of the initial transient.

**Figure 3:**
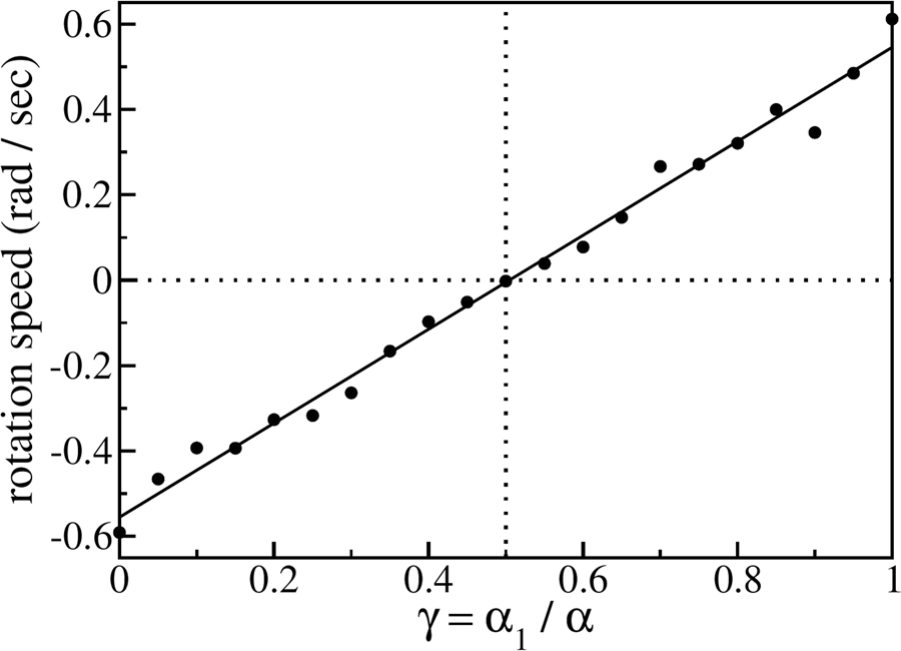
Time-averaged rotation speed $\langle \dot{\varphi }\rangle$ in the ratchet regime for different tooth geometries. The solid line shows a linear regression to the simulation data (circles). The vertical dotted line indicates symmetric teeth (no rotation).

When vibrated, the circular ratchet displays several dynamical regimes. One of the simplest possible ones is a steady rotation, positive or negative as illustrated in Fig. 2. The rotation speed depends on the teeth asymmetry, represented by the ratio *γ* ≡ *α*_1_/*α*. In the simulation shown in Fig. 2 we used *α*_1_/*α* = 0. As seen from the figure, the rotation angle *ϕ* essentially follows a straight line, whose inclination gives the speed of rotation. By varying the ratio *α*_1_/*α* one may tune the magnitude and the sign of the speed. This is illustrated in Fig. 3.

The fact that the circular ratchet starts to rotate when vibrated shows that the particles inside the ratchet exert a net torque on it. To measure this torque we vibrate the ratchet but lock its rotational degree of freedom. This means that only a one-way coupling between the particles and the ratchet device is considered: While the dynamics of the ratchet influences the dynamics of the particles, the particles are not allowed to influence the motion of the ratchet, preventing it from rotating. In other words, the ratchet is mounted in a way that does not allow rotation.

**Figure 4:**
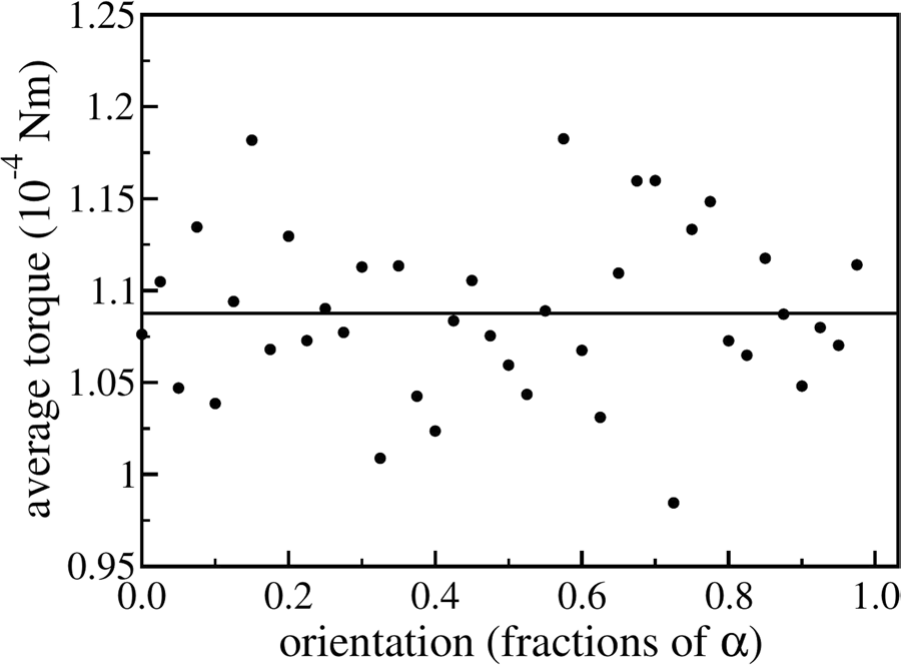
Time-averaged torque on a vibrated but rotationally locked ratchet. The solid line shows a linear regression to the simulation data (circles).

In Fig. 4 the torque exerted by the particles is measured as a function of the rotation angle (i.e. the orientation) of the ratchet for a given tooth profile. Surprisingly, Fig. 4 shows that the torque does not depend on the orientation (the rotation angle) of the ratchet. Especially, it points in the same direction for all orientations of the ratchet and, thus, makes it rotate. More important, the fact that the torque does not depend noticeable on the orientation of the ratchet teeth up to statistical fluctuations, highlights that the ratchet rotation is not an artifact due to unavoidable asymmetries in the initial condition.

The constant driving torque measured for the rotationally locked ratchet suggests, that the freely rotating ratchet would accelerate continuously. However, what one observes is that, after a transient, the ratchet rotates with a constant angular speed. This implies the presence of a second torque, counteracting the driving torque.

**Figure 5:**
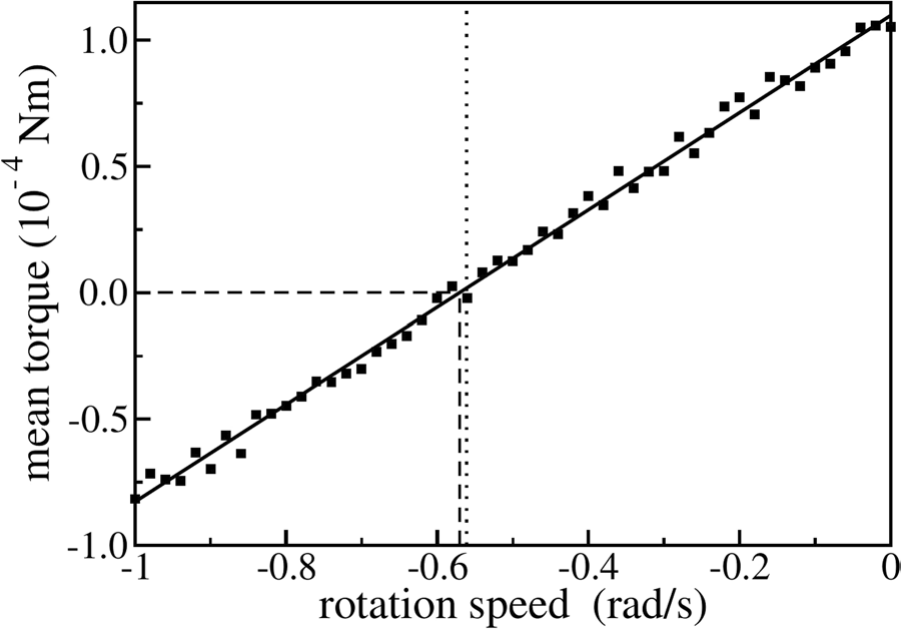
Time-averaged torque on a vibrated and externally rotated ratchet; The solid line shows a linear regression to the simulation data (squares). The dotted line indicates the rotation speed of the corresponding vibrated but freely rotating ratchet (see Fig. 1). The dashed lines highlight the stationary state where driving and breaking torques are in balance.

To determine the torque limiting the angular velocity we vibrate it as before, but additionally enforce rotation at a given angular velocity. Using the forces from the DEM simulations we then measure the torque exerted by the particles on the ratchet. Figure 5 shows this total torque as a function of the angular velocity imposed on the ratchet. This torque contains the constant driving torque (see Fig. 4) together with the decelerating torque and depends linearly on the angular velocity of the ratchet. We conjecture that the vibrated but freely rotating ratchet assumes the angular velocity, where the driving and the decelerating torques are in balance and the total torque, hence, vanishes. Figure 5 corroborates this conjecture and shows that the overall torque vanishes (dashed lines) to good accuracy at the angular velocity of the freely rotating ratchet (dotted line, see further Fig. 2). The decelerating torque results from the fact that once the ratchet rotates, the (dissipative) particles inside it will be dragged along and lifted up by the rough wall. This transport mechanism is limited by the angle of repose and causes, a certain decelerating torque on the ratchet.

Figure 5 implies that this decelerating torque is proportional to the rotation velocity of the ratchet. The dynamics of the ratchet are, therefore, governed by the equation of motion

1 $$I\ddot{\varphi }=M-\beta \dot{\varphi }$$

where *I* = 0.0012 kgm^2^ is the moment of inertia of the rotatable dented cylinder. *M* ≈ 1.09 ⋅ 10^−4^ Nm is the driving torque resulting from the interaction between the particles and the vibrated dented cylinder (see Fig. 4). In the stationary state the ratchet rotates with a constant angular velocity of ${\dot{\varphi }}_{s}\approx -0.56\,{\rm{rad}}/{\rm{s}}$. In this case Eq. 1 simplifies to $M-\beta {\dot{\varphi }}_{s}\,=\,0$ and the friction coefficient is given by $\beta =M/{\dot{\varphi }}_{s}\approx 1.95\cdot {10}^{-4}{{\rm{kgm}}}^{2}/{\rm{s}}$. For the initial conditions *ϕ*(0) = *ϕ*_0_ and $\dot{\varphi }\mathrm{(0)=}{\dot{\varphi }}_{0}$ Eq. 1 has the solution

2 $$\varphi (t)=\frac{I}{\beta }({\dot{\varphi }}_{s}-{\dot{\varphi }}_{0})({e}^{-\frac{\beta }{I}t}-1)+{\dot{\varphi }}_{s}t+{\varphi }_{0}$$

**Figure 6:**
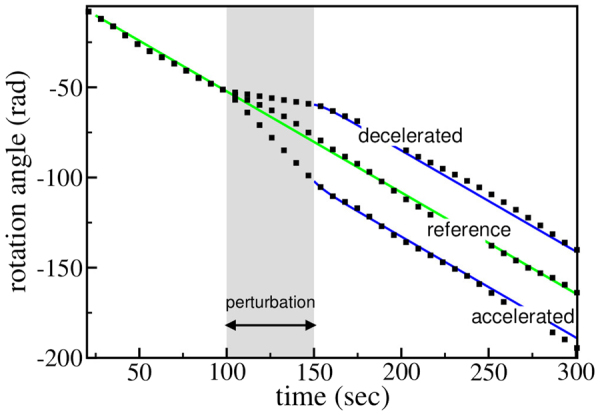
Time dependence of the rotation angle *ϕ* in case of perturbations (temporary accelerated or decelerated rotation). The squares indicate the simulation data. The blue (black) lines show the prediction according to Eq. 2. The parameters used for the perturbation are *ϕ*_0_ = −59.6 rad and ${\dot{\varphi }}_{0}=-0.15\,{\rm{rad}}/{\rm{s}}$  for the deceleration and *ϕ*_0_ = −102.0 rad and ${\dot{\varphi }}_{0}=-1.0\,{\rm{rad}}/{\rm{s}}$ for the accelerated motion. After a short transient, the ratchet returns to its stationary rotation velocity in both cases. It is therefore robust against perturbations of its rotation velocity.

Equation 1 further suggests, that the equilibrium between the driving and the decelerating torque is robust against perturbations. If the freely rotating ratchet is forced to rotate with a velocity higher $(|{\dot{\varphi }}_{0}| > |{\dot{\varphi }}_{s}|)$ or lower $(|{\dot{\varphi }}_{0}| < |{\dot{\varphi }}_{s}|)$ than its equilibrium velocity ${\dot{\varphi }}_{s}$, it will return to the latter in both cases according to Eq. 2. Figure 6 illustrates this behavior.

**Figure 7:**
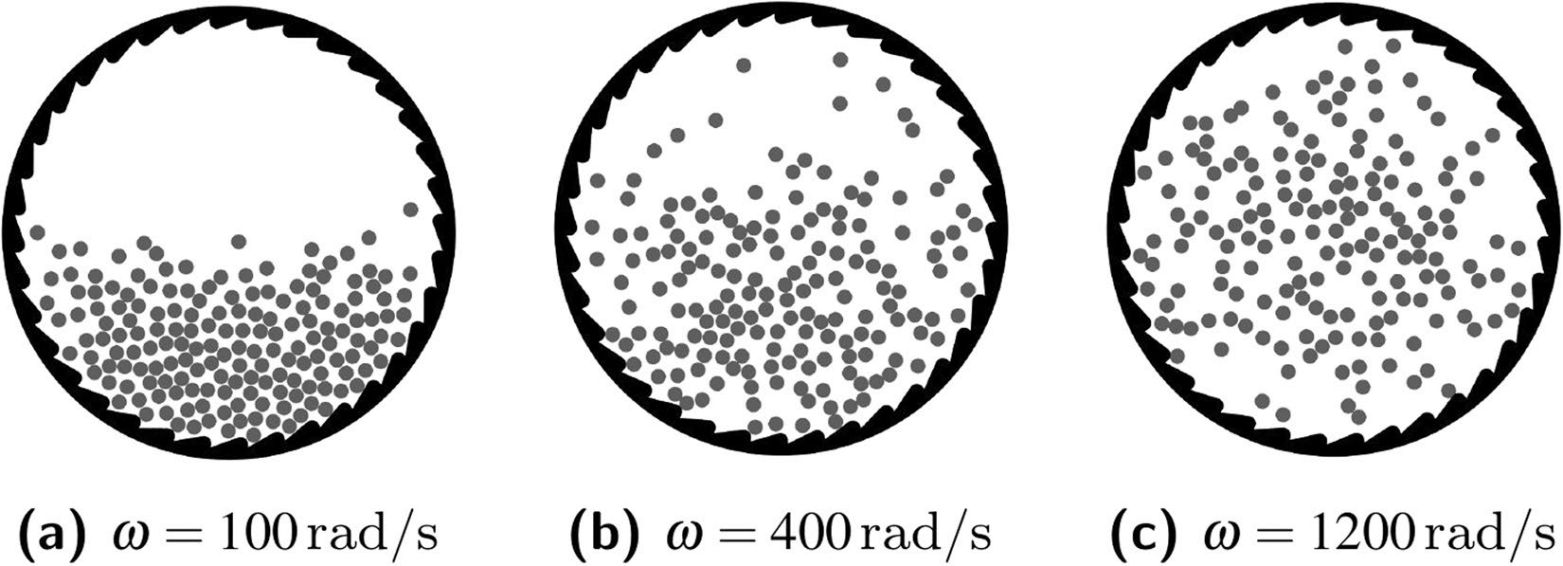
Characteristic dynamic regimes for different driving frequencies. (**a**) low frequency, excited sediment; (**c**) high frequency, fluidized; (**b**) intermediate frequency, transition region.

So far we studied the ratchet for driving parameters which allow for a certain excitation of the particles but operating in a situation where gravity limits their motion to the bottom of the ratchet (see e.g. Fig. 7(a)). By significantly changing the driving parameters (amplitude and frequency) we, of course, expect different dynamic regimes.

**Figure 8:**
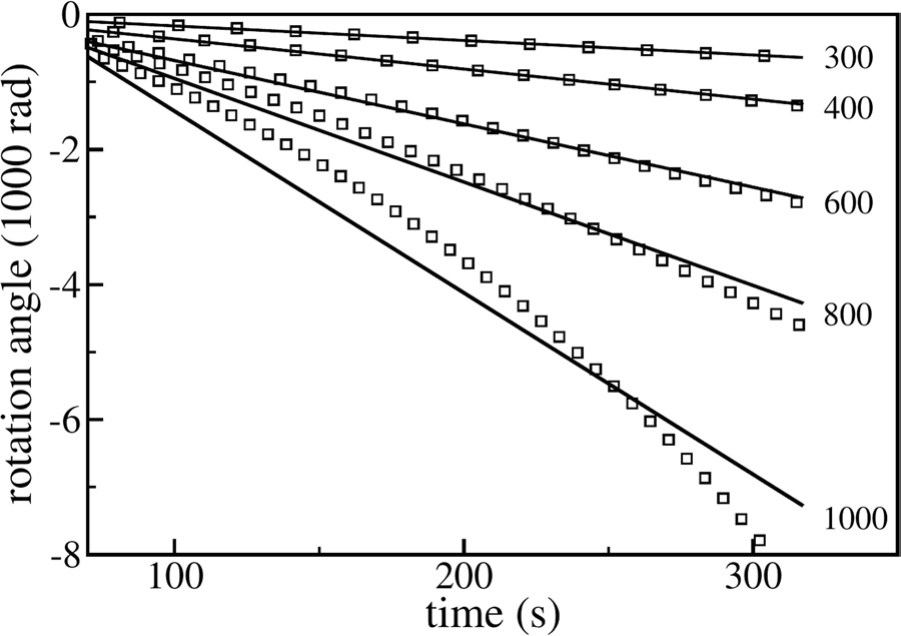
Breakdown of the decelerating mechanisms for high driving frequency. The symbols show the rotation angle as a function of time for different driving frequencies indicated by the labels, the solid lines show the corresponding linear regression in each case.

**Figure 9:**
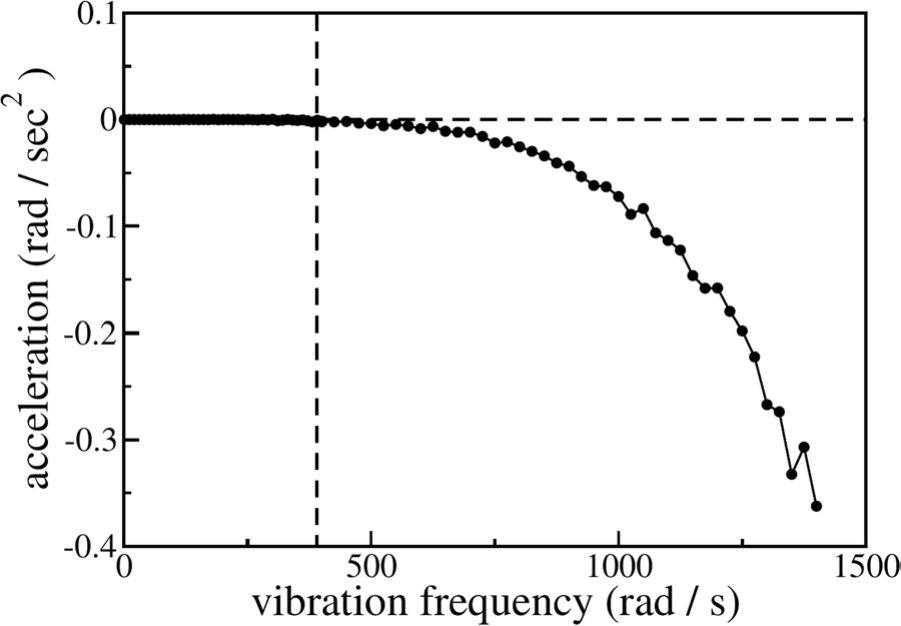
The acceleration of the rotation angle $\ddot{\varphi }(t)$ as a function of time. The vertical dashed line clearly separates two dynamic regimes: For driving frequencies $\omega \,\lessapprox \,400\,{{\rm{s}}}^{-1}$ gravity dominates, for larger frequencies the granulate enters a fluidized state (see text).

**Figure 10:**
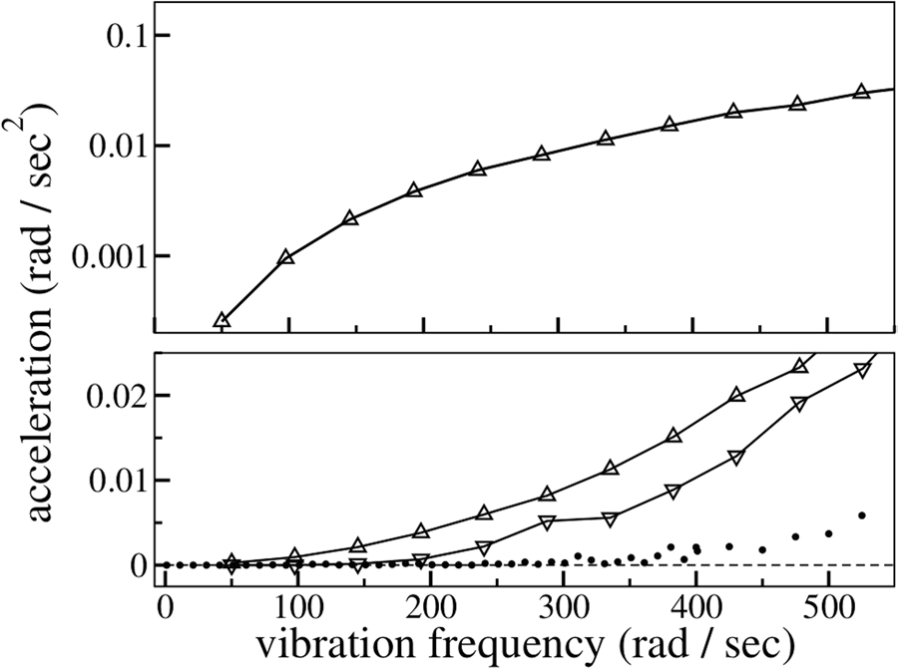
Acceleration of the rotation angle as a function of the vibration frequency for different magnitudes *g* of gravity. Lower panel: *g* = 9.81 m/*s* ^2^ (circles), *g* = 4 m/*s* ^2^ (triangles pointing downwards), *g* = 0 m/*s* ^2^ (triangles pointing upwards). Upper panel: *g* = 0 m/*s* ^2^, logarithmic representation.

First, we vary the driving frequency *ω* and measure the rotation angle *ϕ*. Figure 8 shows *ϕ* as a function of time for five driving frequencies. As seen from this figure, for $\omega \,\gtrapprox \,400\,{{\rm{s}}}^{-1}$, the rotation angle *ϕ*(*t*) may no longer be well-fitted by a straight line, implying that the ratchet rotation speed is not constant. In fact, the rotation speed gets larger and larger, indicating that in the fluidized regime, for high driving frequencies, the decelerating mechanisms break down gradually. The remaining (now dominating) driving torques accelerate the rotation of the ratchet. Measuring the average rotation speed is, of course, meaningless in this regime. For this reason, in Fig. 9, we plot the acceleration of the rotation angle, $\ddot{\varphi }(t)$. In this figure, the dashed vertical line at *ω* ≈ 400 s^−1^ clearly separates two markedly different dynamical regimes. For $\omega \,\lessapprox \,400\,{{\rm{s}}}^{-1}$ gravity dominates, leading to the sediment-like pattern shown in Fig. 7(a). For higher driving frequencies, the granulate enters a fluidized gaseous state as shown in Fig. 7(b) and (c). Figure 10 (lower panel) further shows that the driving frequency at which the decelerating mechanisms and, thus, the stationary state breaks down, is shifted to smaller values as the magnitude of gravity is decreased. This is expected as a granulate in the presence of gravity *g* which is vibrated sinusoidally in vertical direction with the amplitude *a* and frequency *ω* enters the gas-like fluidized state once the dimensionless shaking acceleration Γ = *aω* ^2^ */ g* exceeds a certain system specific value which is typically sharply larger than one^15^. Under conditions of weightlessness there is no such transition between a fluidized and a sediment-like regime and, thus, no stationary state in which the ratchet rotates at constant angular velocity. Instead, the acceleration of the rotation is always finite and decreases continuously as the vibration frequency is reduced (see upper panel of Fig. 10).

**Figure 11:**
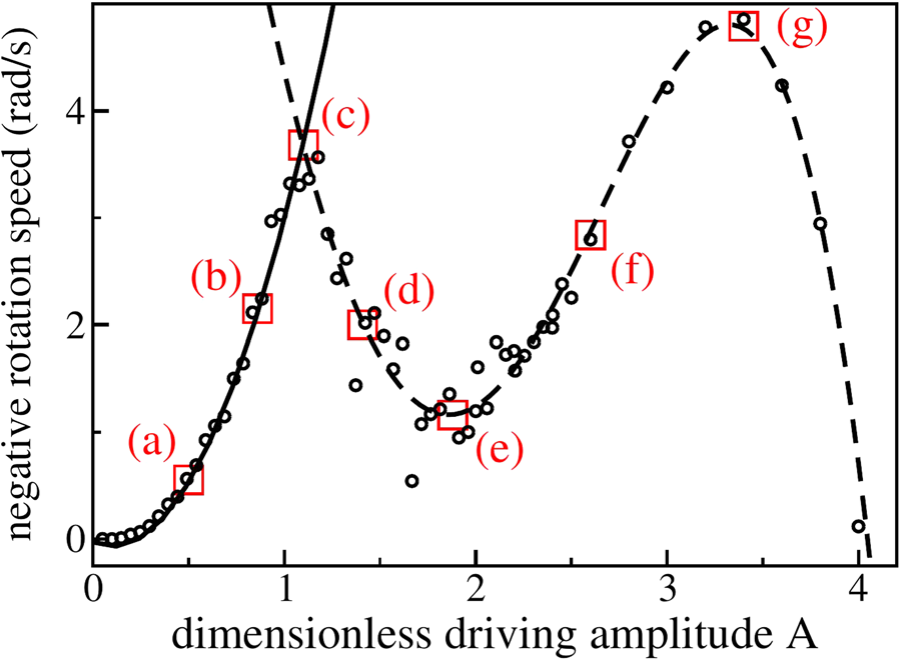
The symbols show the (negative) rotation speed as a function of the dimensionless driving amplitude (*A*). The solid line is a quadratic fit to the data left to the first minimum at *A* ≈ 1.1. The dashed line shows a fourth order fit to the data right of the first minimum. Figure 12 shows flow fields for the amplitudes indicated by the squares. The labels denote the corresponding panels in Fig. 12.

**Figure 12:**
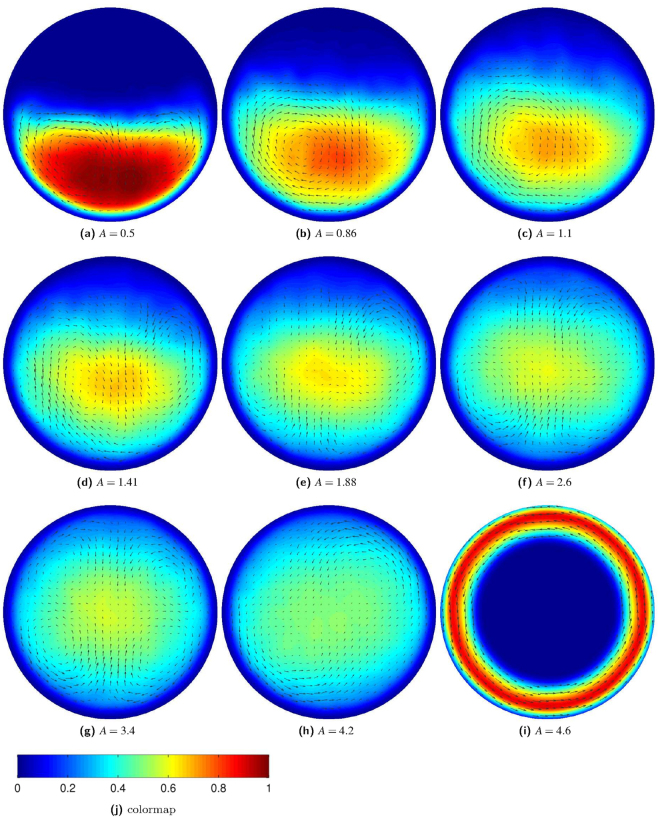
From left to right and bottom to top: fields of density and vectorial velocity for increasing driving amplitudes. Note that the amplitudes indicated in the subcaptions are measured in units of particle diameters (*A* = *a*/*d*) and that the density fields in the background are normalized to the maximum density. Panel (j) shows the color scheme used to visualize the fields of density. The length of the arrows indicating the velocities is scaled in a way that the individual arrows just do not overlap for each image to improve visibility.

In the next experiment, the average rotation speed is measured when the driving amplitude is varied at constant frequency. The result is shown in Fig. 11. The complicated structure of the curve can be understood by investigating the corresponding flow fields (see Fig. 12) which reveal at least three characteristic dynamical regimes. Note that the driving amplitudes indicated are measured in units of particle diameters, $A=a/d$. Starting from small amplitudes, we observe two convection rolls which almost degenerate to a single convection roll at the first maximum of the average rotation speed $|\langle \dot{\varphi }\rangle |$ at *A* ≈ 1.1. As indicated by the density fields in Fig. 12, gravity dominates in this regime and concentrates the particles into the lower part of the ratchet. For higher amplitudes, the convection rolls become less prominent and the influence of gravity decreases. Starting from the minimum of the $|\langle \dot{\varphi }\rangle |(A)$ curve at *A* ≈ 1.88, the material begins to slosh back and forth between the ratchet bottom and the top. This sloshing motion is characterized by four convection rolls which drive the ratchet effectively, leading to the second maximum in $|\langle \dot{\varphi }\rangle |(A)$ at *A* ≈ 3.4. For yet higher amplitudes (*A* > 4.3) the sloshing motion becomes unstable. Eventually, the whole material collapses and sticks to the (rough) wall resulting in a constant rotation velocity corresponding to the driving frequency ($A\,\geqq \,4.6$).

## Conclusions and outlook

We performed a systematic numerical study of the dynamical characteristics of a circular ratchet whose teeth may be continuously tuned to impart negative and positive angular velocities to the ratchet. We described the interplay of two counteracting torques underlying the circular ratchet operation and performance. Circular ratchets display three different dynamical regimes, imposed by the external periodic drive, that we refer to as low-frequency excited regime, the intermediate transitional regime, and the fluidized high-frequency regime. We also found two marked distinct behaviors of the acceleration of the rotation angle at high driving frequencies. Initially, for lower frequencies, we find gravity to dominate the dynamics while beyond a certain threshold the granulate enters a fluidized state, occupying the whole space available, not just the bottom of the ratchet, as is the case when gravity dominates. Keeping the driving frequency constant, we also investigated what happens with the ratchet when the amplitude of the drive is varied. In this case the rotation velocity displays a complex pattern which, however, may be understood in terms of the flow fields present in the ratchet. All the aforementioned regimes were found to be robust and reproducible. In summary, as it is clear from our analysis, circular ratchets involve a number of parameters that still remain to be explored. However, we hope that the complexity of the dynamical regimes found may motivate further exploration of this interesting device.

## Methods

Here we describe the numerical methods used in this work. When not specified otherwise, the parameters indicated in Table 1 have been used. We wish to mention that the described dynamical regimes are not an artifact of the chosen parameters. Qualitatively similar behavior can be found for other geometries, different number of particles, other materials and driving parameters. Similarly, the described results do not depend on the details of the short range repulsive particle-particle interaction assumed for the simulations, as only the transfer of momentum between the particles and the dented cylinder is relevant. For instance ideal hard spheres would lead to qualitatively similar results. Especially friction between particles does not influence the results qualitatively and was, therefore, neglected in the simulations.

**Table 1:** Default simulation parameters. Note that the specified dissipative parameter *A*_dis_ for the interaction of two particles corresponds to the coefficient of restitution *ε* = 0.75 for the impact velocity 3m/s.

symbol	value	unit	meaning
*d*	0.005	m	particle diameter
*r*	15 *d*	m	ratchet radius
*h*	*d*	m	tooth height
*α*	*π*/18	rad	see Fig. 1
*d*_*w*_	0.5 *d*	m	plate thickness
*γ* ≡ *α*_1_/*α*	0	—	see Fig. 1
*N*	169	—	number of particles, filling fraction ≈0.2
*A* ≡ (*a*)/(*d*)	0.5	—	dimensionless driving amplitude
*ω*	198	rad/s	driving frequency
*g*	9.81	m/s^2^	gravitational acceleration
*ρ*_*p*_	2000	kg/m^3^	density particle (soft rubber)
*ρ*_*r*_	1190	kg/m^3^	density container (acryl)
*Y*	0.01	10^9^N/m^2^	Young’s Modulus particle
*ν*	0.5	—	Poisson’s ratio particle
*A*_dis_	1.93 ⋅ 10^−5^	s	dissipative parameter (see Eq. 3)

### Discrete element method (DEM)

The dynamics of the circular ratchet shown in Fig. 1 was simulated using the discrete element method (DEM). DEM is a well-known method used for the numerical simulation of systems consisting of many solid macroscopic objects. The dynamics of these objects are described by classical mechanics: If all acting forces are known, the temporal evolution of the system may be described by Newton’s equations of motion. In general each of the objects has three translational and three rotational degrees of freedom. The dynamics of a system of *n* macroscopic solid objects are, therefore, governed by 6*n* coupled ordinary differential equations (ODEs). The simulation method corresponding to the solution of this system of ODEs is often referred to as DEM. This method dates back to works by Rahman and Verlet^16,17^, where it has been used to study the physics of simple fluids. Subsequently, motivated by other applications^18–21^, the method was later adapted to deal with granular systems. A comprehensive survey of the method can be found in e.g.^22^. Due to its historic origin, DEM is also often referred to as (force-based) molecular dynamics (MD).

### Particle-particle-interaction

The particles inside the circular ratchet are modeled as frictionless viscoelastic spheres. Further we consider a two dimensional simulation where the motion of the particles is restricted to the plane defined by the dented circle (see Fig. 1). The contact between two frictionless viscoelastic spheres *i* and *j* with radii *R*_*i*_ and *R*_*j*_ located at positions $\overrightarrow{r}$_*i*_ and $\overrightarrow{r}$_*j*_ is described by the interaction force

3 $$F={F}^{{\rm{el}}}+{F}^{{\rm{dis}}}={\rho }_{{\rm{el}}}{\xi }^{3/2}-\frac{3}{2}{A}_{{\rm{dis}}}\,{\rho }_{{\rm{el}}}\dot{\xi }\sqrt{\xi }\,,$$

where

4 $${\rho }_{{\rm{el}}}\equiv \frac{2Y\sqrt{{R}_{{\rm{eff}}}}}{\mathrm{3(1}-{\nu }^{2})}\,\quad {\rm{and}}\quad \xi \equiv {R}_{i}+{R}_{j}-|{\overrightarrow{r}}_{j}-{\overrightarrow{r}}_{i}|,$$

where *Y*, *ν* and *R*_eff_ denote the Young’s modulus, the Poisson’s ratio and the effective radius *R*_eff_ = *R*_*i*_ *R*_*j*_/(*R*_*i*_ + *R*_*j*_), respectively. *ξ* is defined as the mutual compression of two particles in contact. The elastic part *F* ^el^ of this widely used collision model^23–25^ is given by the familiar Hertz contact force^26^. The dissipative part, *F* ^dis^, was first motivated in ref.^27^ and then rigorously derived in refs^28,29^ Note that only the approach in ref.^28^ leads to an analytic expression for the parameter *A*_dis_ as a function of the elastic and viscous material parameters. Equation 3 is then used to compute the forces acting on the particles which are, in turn, needed to solve Newton’s equations of motion.

### Particle-wall interaction

The particle-wall interaction is modeled similar to the particle-particle interaction. When a particle impacts the wall (the dented cylinder), we consider a virtual particle which is located such that the contact point between the virtual and the physical particle is located exactly at the position where the physical particle impacts the wall. The material parameters and the radius of the virtual particle is chosen identical to the physical particles and the interaction force between the physical and the virtual particles is then computed according to the interaction force, Eq. 3. Because there is no model for the interaction force between a viscoelastic sphere and a sharp edge, we do not consider a sharp edged sawtooth profile (dashed line in Fig. 1). Instead we smoothen the wall of the ratchet by sweeping a sphere (diameter *d*) along the sharp edged saw teeth, resulting in the ratchet profile shown in Fig. 1. The dented cylinder is mounted in a way such that it can only rotate around its axis of symmetry and such that translation is only possible in the vertical direction. Vertical sinusoidal driving is then used to excite this remaining translational degree of freedom. The interaction i.e. the contact between the dented cylinder and a particle hence causes a torque which excites the remaining rotational degree of freedom of the ratchet. The two circular plates of thickness *d*_*w*_ which enclose the dented cylinder as shown in Fig. 1 would be relevant to confine the particles in the corresponding physical experiment as discussed in^14^. For the 2D simulations presented in this work, they are not explicitly relevant. They are only considered to compute the mass and the moment of inertia of the ratchet device, where the material parameters indicated in Table 1 are used.

### Video

The video mentioned in the text is available at www.mss.cbi.fau.de/sup/ratchet_dynamics.mp4.

## Electronic supplementary material

Supplementary Video File

## References

[1] Hänggi P, Marchesoni F (2009). Artificial Brownian motors: Controlling transport on the nanoscale. Rev. Mod. Phys..

[2] Hänggi, P. & Bartussek, R. In *Nonlinear Physics and Complex Systems - Current Status andFuture Trends*, edited by J. Parisi *et al*., Lecture Notes in Physics (Springer, Berlin, 1996), Vol. 476, pp. 294–308.

[3] Kay ER, Leigh DA, Zerbetto F (2007). Synthetische molekulare Motoren und mechanische Maschinen. Angew. Chem..

[4] Reimann P (2002). Brownian motors: noisy transport far from equilibrium. Phys. Rep..

[5] Cubero, D. & Renzoni, F. Brownian Ratchets: From Statistical Physics to Bio and Nano-motors, Cambridge University Press, Cambridge (2016)

[6] Jarzynski C, Mazonka O (1999). Feynman’s ratchet and pawl: An exactly solvable model. Phys. Rev. E.

[7] Kulish O, Wright AD, Terentjev EM (2016). F1 rotary motor of ATP synthase is driven by the torsionally-asymmetric drive shaft. Scientific Reports.

[8] Jülicher F, Ajdari A, Prost J (1997). Modeling molecular motors. Rev. Mod. Phys..

[9] Jülicher F, Prost J (1995). Cooperative Molecular Motors. Phys. Rev. Lett..

[10] Smoluchowski M (1912). Experimentell nachweisbare, der üblichen Thermodynamik widersprechende Molekularphänomene. Phys. Z..

[11] Feyman, R. P., Leighton, R. B. & Sands, M. *The Feynman Lectures on Physics*, (Addison-Wesley, Reading, 1963), vol. 1, Chap. 46.

[12] The work of Feynman^12^ is reviewed critically by J.M.R. Parrondo and P. Español, Criticism of Feynman’s analysis of the ratchet as an engine, Am. J. Phys. **64**, 1125 (1996).

[13] Ang YS, Ma Z, Zhang C (2015). Quantum ratchet in two-dimensional semiconductors with Rashba spin-orbit interaction. Scientific Reports.

[14] Heckel M, Müller P, Pöschel T, Gallas JAC (2012). Circular ratchets as transducers of vertical vibrations into rotations. Phys. Rev. E.

[15] Eshuis P, van der Weele K, van der Meer D, Bos R, Lohse D (2007). Phase diagram of vertically shaken granular matter. Phys. Fluids.

[16] Verlet L (1967). Computer experiments on classical fluids. I. Thermodynamical properties of Lennard-Jones molecules. Phys. Rev..

[17] Rahman A (1964). Correlations in the motion of atoms in liquid Argon. Phys. Rev..

[18] Gallas JAC, Herrmann HJ, Sokołowski S (1992). Convection cells in vibrating granular media. Phys. Rev. Lett..

[19] Haff PK, Werner BT (1986). Computer Simulation of the meachnical sorting of grains. Powder Technology.

[20] Walton OR, Braun RL (1986). Viscosity, granular-temperature, and stress calculations for shearing assemblies of inelastic, frictional disks. J. Rheol..

[21] Cundall PA, Strack O (1979). A discrete numerical model for granular assemblies. Géotechnique.

[22] Pöschel, T. & Schwager, T. Computational Granular Dynamics, Springer, Berlin (2005).

[23] Kruggel-Emden E, Simek H, Rickelt S, Wirtz S, Scherer V (2007). Review and extension of normal force model for the discrete element method. Powder Technol..

[24] Stevens AB, Hrenya CM (2005). Comparison of low Reynolds number k-e turbulence models in predicting fully developed pipe flow. Powder Technol..

[25] Schäfer J, Dippel S, Wolf DE (1996). Force schemes in simulation of granular materials. J. Phys. I France.

[26] Hertz H (1882). Über die Berührung fester elastischer Körper. J. reine und angewandte Mathematik.

[27] Kuwabara G, Kono K (1987). Restitution coefficient in a collision between two spheres. Jap. J. Appl. Phys.

[28] Brilliantov NV, Spahn F, Hertzsch J-M, Pöschel T (1996). Model for collisions in granular gases. Phys. Rev. E.

[29] Morgado WAM, Oppenheim I (1997). Energy dissipation for quasielastic granular particle collisions. Phys. Rev. E.

